# Genetic diversity and population structure of Mongolian regional horses with 14 microsatellite markers

**DOI:** 10.5713/ab.21.0497

**Published:** 2022-03-02

**Authors:** Jihye Yun, Baatartsogt Oyungerel, Hong Sik Kong

**Affiliations:** 1 Department of Applied Biotechnology, The Graduate School of Hankyong National University, Anseong 17579, Korea; 2 School of Animal Science and Biotechnology, Mongolian University of Life Sciences, Zaisan 17024, Ulaanbaatar, Mongolia; 3 Genomic Informatics Center, Hankyong National University, Anseong 17579, Korea; 4 Gyeonggi Regional Research Center, Hankyong National University, Anseong 17579, Korea

**Keywords:** Genetic Diversity, Horse, Microsatellites, Mongolia, Relationships, Structure

## Abstract

**Objective:**

This study aimed to identify the genetic diversity and population structure of Mongolian horse populations according to the province of residence (Khentii, KTP; Uvs, USP; Omnogovi and Dundgovi, GOP; Khovsgol, KGP) using 14 microsatellite (MS) markers.

**Methods:**

A total of 269 whole blood samples were obtained from the four populations (KTP, USP, GOP, KGP) geographically distinct provinces. Multiplex polymerase chain reaction (PCR) was conducted using 14 MS markers (AHT4, ASB2, ASB17, ASB23, CA425, HMS1, HMS2, HMS3, HMS6, HMS7, HTG4, HTG6, HTG7, and VHL20), as recommended by the International Society for Animal Genetics. Capillary electrophoresis was conducted using the amplified PCR products, alleles were determined. Alleles were used for statistical analysis of genetic variability, Nei’s DA genetic distance, principal coordinate analysis (PCoA), factorial corresponding analysis (FCA), and population structure.

**Results:**

On average, the number of alleles, expected heterozygosity (H_Exp_), observed heterozygosity (H_Obs_), and polymorphic information content among all populations were 11.43, 0.772, 0.757, and 0.737, respectively. In the PCoA and FCA, GOP, and KGP were genetically distinct from other populations, and the KTP and USP showed a close relationship. The two clusters identified using Nei’s DA genetic distance analysis and population structure highlighted the presence of structurally clear genetic separation.

**Conclusion:**

Overall, the results of this study suggest that genetic diversity between KTP and USP was low, and that between GOP and KGP was high. It is thought that these results will help in the effective preservation and improvement of Mongolian horses through genetic diversity analysis and phylogenetic relationships.

## INTRODUCTION

Horses (*Equus caballus*) have been domesticated in steppes and have played an important role in the development of several civilizations. In Mongolia, horses are one of the five livestock species, and according to [1], as of 2020, of the approximately 67.07 million livestock in Mongolia, 4.09 million are horses. The Mongolian horse is one of the most ancient breeds in the world and has adapted to Mongolia’s climate and environment. The Mongolians were able to move easily using horses to find better grasslands, increase the number of livestock, and expand foreign relations. Therefore, Mongolian horses occupy an important place in Mongolia’s nomadic lives.

The ancient wild Mongolian horse is known as Przewalskii [2,3]. Though the ancient Mongolian horse may have been the ancestor of several modern horse breeds, the protection and management of Mongolian horse’s genetic resources is still insufficient. As the importance of livestock genetic resources increases worldwide, various studies are being conducted on the molecular genetic properties of genetic resources in order to preserve their value. Studies on genetic diversity are essential for the management of genetic resources through genetic characteristic analysis, breeding, and discovery of useful traits.

Microsatellites (MS) are used as molecular markers for genetic diversity analysis and breeding programs [4,5]. These molecular markers can be used to obtain information on the phylogenetic relationships or genetic variation. Microsatellite markers were first characterized in horses by Ellegren et al [6], Marklund et al [7]. These have since been used extensively to investigate the genetic diversity and breed characteristics of horse populations. As some horse breeds were domesticated in the steppes of Mongolia, it was predicted that there would be a high genetic diversity [8]. However, genetic analysis using MS markers is yet to be conducted among Mongolian horses. To study livestock genetic resources in Mongolia, it is necessary to analyze the genetic diversity and genetic relationship between Mongolian horse populations using an efficient MS marker.

In Mongolia, various breeds of horses inhabit the climate and topographical characteristics of each province. According to Wright [9] and Ganbold et al [10], Mongolian horses mainly inhabit steppes and dry areas such as Gobi. The appearance of Mongolian horses varies greatly depending on their habitat. Genetic characteristics also appear to be different. Khentii Province is a grassland region located in the eastern part of Mongolia, where various plants grow naturally. The Uvs province is located in the west of Mongolia, and the Omnogovi and Dundgovi provinces comprise the steppes in southern Mongolia. The Khovsgol province is a pasture located in northern Mongolia and has a cold climate.

As such, Mongolian horses have different traits depend ing on the province where they live, but the genetic diversity and correlations using MS markers have not yet been verified. Therefore, this study aimed to identify the genetic characteristics of Mongolian horses according to the province of residence using 14 MS markers published by the International Society of Animal Genetics (ISAG). We analyzed the genetic relationships and genetic diversity of horses raised in each of the five provinces (Khentii, Uvs, Omnogovi, Dundgovi, and Khovsgol) in Mongolia, which were divided into four populations. Genetic diversity analysis is expected to help in the effective protection and management of Mongolian horse genetic resources. In addition, the genetic structure and phylogenetic relationships of the Mongolian horse populations were also identified, and can be used as basic data for research on genetic resources of not only horses but also other livestock in Mongolia.

## MATERIAL AND METHODS

### Genomic DNA extraction

**Figure 1:**
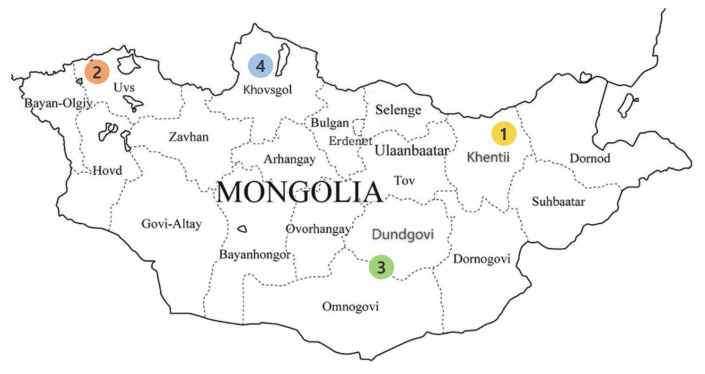
Four Mongolian horse populations from the five provinces of Mongolia. 1. Khentii province (KTP); 2. Uvs province (USP); 3. Omnogovi and Dundgovi province (GOP); and 4. Khovsgol province (KGP).

The experimental methods were approved by the Hankyong National University Animal Ethics Committee, Anseong, Republic of Korea (No.2021-1). A total of 269 whole blood samples were obtained from the four populations of the five geographically distinct provinces (Table 1, Figure 1). Sampling sites included i) East Mongolia Khentii Province (KTP, n = 53); ii) West Mongolia Uvs Province (USP, n = 43); iii) Southern Mongolia Omnogovi and Dundgovi provinces (GOP, n = 133); and iv) Northern Mongolia Khovsgol province (KGP, n = 40). Genomic DNA was extracted from blood samples using the methods described for QuickGene 810 (Kurabo, Osaka, Japan). The concentration and purity of the extracted genomic DNA were evaluated using an ND-1000 UV-Vis spectrophotometer (NanoDrop Technologies, Wilmington, DE, USA).

### Information on the microsatellite markers

The genetic diversity of Mongolian horse populations was identified using 14 MS markers (AHT4, ASB2, ASB17, ASB23, CA425, HMS1, HMS2, HMS3, HMS6, HMS7, HTG4, HTG6, HTG7, and VHL20), as recommended by the ISAG.

### Composition of multiplex-polymerase chain reaction and the polymerase chain reaction procedure

Multiplex polymerase chain reaction (PCR) was conducted using the Equine Genotypes Panel 1.1 Kit (Thermo Fisher Scientific, Waltham, MA, USA) for genotyping of the 14 MS markers. Following the manufacturer’s instructions, the reaction mixture was prepared using 2 μL genomic DNA (1.0 ng/μL), 9 μL master mix, and 9 μL primer mix, obtaining a total volume of 20 μL. The PCR was conducted using a GeneAmp PCR system 9700 (Applied Biosystems, Waltham, MA, USA). The PCR amplification was conducted using the following conditions: pre-denaturation at 98°C for 3 min followed by 30 cycles of 15 s at 98°C, 75 s at 60°C, and 1 cycle of 30 s at 72°C. The final extension step was performed at 72°C for 5 min and the mixture was cooled to 4°C.

### Genotyping of microsatellite

Using Hi-Di formamide, amplified PCR products were diluted 50 to 100 times, and the diluted PCR products (1 μL) were further diluted using a mixture of 9 μL Hi-Di formamide and GeneScan 500LIZ size standard (Applied Biosystems, USA). After denaturation at 95°C for 3 min, capillary electrophoresis was conducted using an ABI 3730xl Genetic Analyzer (Applied Biosystems, USA). The size of each MS marker was determined using GeneMapper ver 5.0 (Applied Biosystems, USA). The determined alleles were collated using Microsoft Excel (Microsoft, Redmond, WA, USA) and used for statistical analysis.

### Statistical analysis of data

The MS Toolkit software [11] was used to calculate the number of alleles, expected and observed heterozygosity (H_Exp_ and H_Obs_, respectively), and polymorphism information content (PIC) values. To identify genetic correlations between populations, allele frequencies for each marker were confirmed using GenAlEx 6.4 [12] and Genetix software [13]. For this, principal coordinate analysis (PCoA) was conducted using GenAlEx 6.4 to display the two-dimensional coordinates. Factorial component analysis (FCA) was conducted using Genetix to display the three-dimensional coordinates. The genetic distance between populations was calculated based on allele frequencies according to Nei’s DA genetic distance [14] using POPTREE2 [15]. A phylogenetic tree was estimated from the genetic distances using the neighbor-joining method [16] with the DISPAN program [17]. Population structure [18,19] was used to estimate the uniformity of the population, and the K value was set to estimate the number of distinct populations (ΔK). To calculate the average estimate and standard deviation of each K value, the length of the burn-in period and the number of Markov chain Monte Carlo (MCMC) repetitions after the burn-in frequency were set, and the optimal K value and genetic uniformity for each cluster were calculated The results were applied to the Structure harvester [20] using the Evanno method [21].

## RESULTS

### Microsatellite polymorphism

The number of alleles, H_Exp_, H_Obs_, and PIC values for the Mongolian horse populations are summarized (Table 2). A total of 160 different alleles were detected, ranging from 6 (HTG7) to 22 (ASB2). H_Exp_ and H_Obs_ ranged from 0.572 (HTG4) to 0.885 (ASB17) and 0.561 (HTG4) to 0.868 (ASB17), with mean values of 0.772 and 0.757, respectively. PIC values ranged from 0.535 (HTG4) to 0.866 (ASB17), with a mean value of 0.737. Except for the HTG4 marker (H_Exp_ = 0.572, PIC = 0.535) used in this study, all other MS markers were considered to be highly useful in analyzing polymorphism of Mongolian horse. MS markers were previously used to assess the genotypic diversity of heterozygosity and PIC value during animal breed selection. According to Botstein et al [22], polymorphisms of MS markers are determined using the following criteria: if the sum of H_Exp_ is ≥0.6 and PIC is ≥0.5, then the marker is determined to be highly polymorphic. The heterozygosity (H_Exp_ and H_Obs_) and PIC values with respect to the four Mongolian horse populations are summarized (Table 3). Among the provinces, the values for mean number of alleles (MNA), H_Exp_, H_Obs_, and PIC were highest for KTP horses (8.57, 0.787, 0.789, and 0.751, respectively) and lowest for KGP populations (7.07, 0.751, 0.738, and 0.706, respectively).

### Genetic distance and phylogenetic analysis

**Figure 2:**
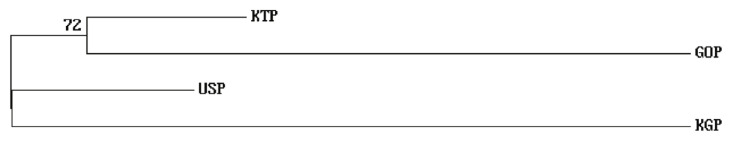
Neighbor-Joined tree showing the genetic distances among the four populations using Nei’s DA genetic distance on the basis of allele frequencies from the 14 MS loci. The number in the branch indicates the percentage of occurrence after 1,000 bootstrap replicates. MS, microsatellite; KTP, Khentii province; USP, Uvs province; GOP, Omnogovi and Dundgovi province; KGP, Khovsgol province.

The genetic divergences among the four Mongolian horse populations based on allele frequencies were calculated according to Nei’s DA genetic distance [16]. The phylogenetic relationships among these Mongolian horse populations were determined using the neighbor-joining tree (Figure 2). Grouping values were determined by 1,000 repetitive “bootstrap” tests to determine the reliability of the neighbor-joining tree and were specified at the branching points of the tree. The smaller the estimated genetic distance is, the closer each population is genetically. In Nei’s DA genetic distance, the KTP and USP populations were the closest (DA = 0.0535), and the largest difference was calculated for the GOP and KGP populations (DA = 0.1797) (Supplementary Table S1). In the phylogenetic tree, the Mongolian horse populations was mainly divided into two clusters. The KTP and GOP populations formed the first cluster, and the KGP population formed the second cluster. The USP breed was located between the two clusters. The division of the populations into two distinct clusters highlighted the presence of clear genetic separation between each province.

### Principal coordinates analysis and factorial component analysis

**Figure 3:**
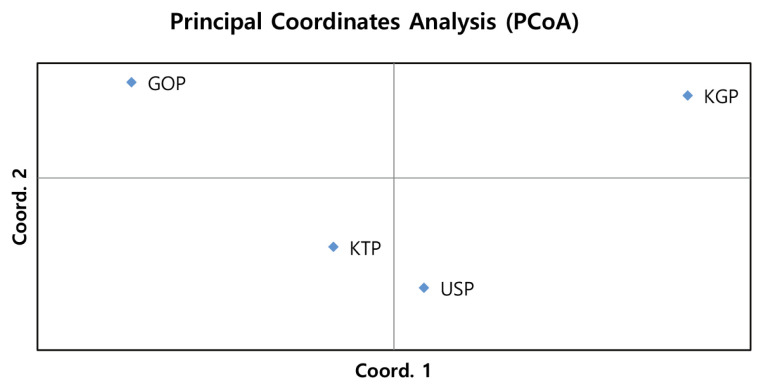
Principal Coordinate Analysis of allele frequencies from the 14 MS loci genotypes in four populations using GenAIEx. MS, microsatellite; KTP, Khentii province; USP, Uvs province; GOP, Omnogovi and Dundgovi province; KGP, Khovsgol province.

**Figure 4:**
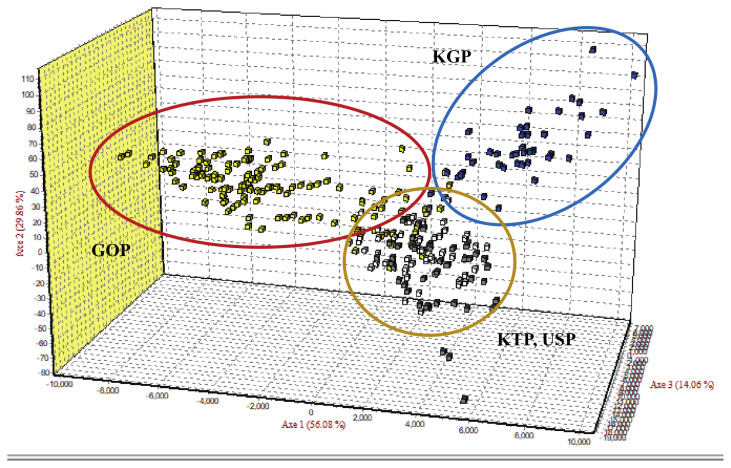
Factorial correspondence analysis of allele frequencies from the 14 MS loci genotypes in four populations using Genetix. MS, microsatellite; KTP, Khentii province; USP, Uvs province; GOP, Omnogovi and Dundgovi province; KGP, Khovsgol province.

As the phylogenetic tree may not take into account the effects of admixture among the four populations, we conducted PCoA and FCA, using allele frequencies of the 14 MS markers, as an alternative approach to understand the genetic relationships among populations. The PCoA contributed to 100% of the variation, including the third ingredient. The first three principal coordinates represented 60.13%, 29.75%, and 10.12% of the total variation, respectively. The KGP and GOP populations were distinct from the other populations (Figure 3). In contrast, the KTP and USP populations were located between the closest genetic distances. The FCA analysis revealed that the three dimensions contributed to a total of 100 with Axis 1 (56.08), Axis 2 (29.86), and Axis 3 (14.06). The KTP and GOP populations were genetically differentiated from other populations and showed a close relationship among certain populations, including KTP and USP (Figure 4). Thus, results of the PCoA analysis were consistent with those of the FCA.

### Uniformity of horse population

**Figure 5:**
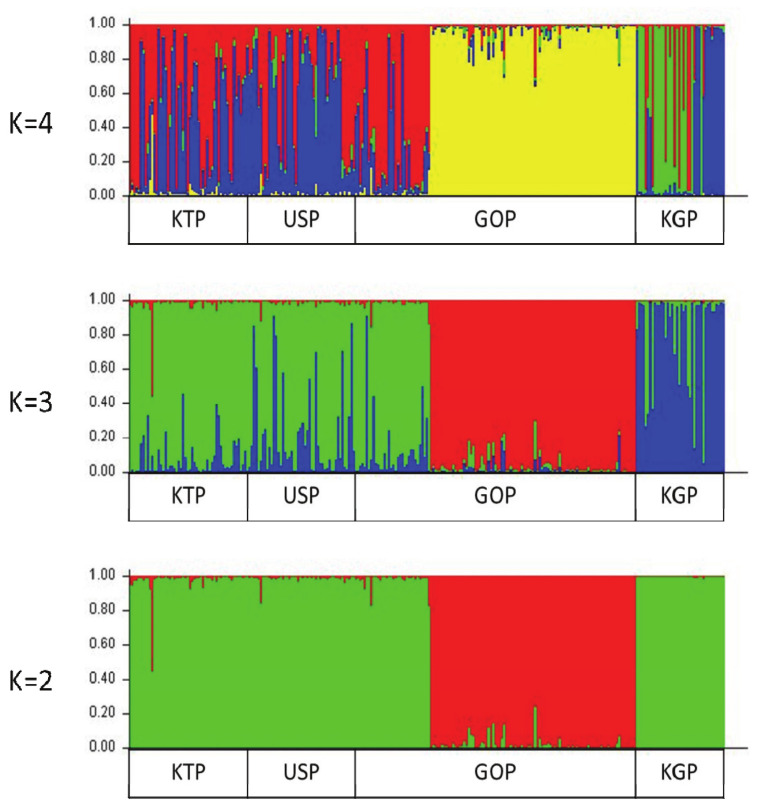
Structure analysis of the four horse populations. Cluster results from a structure analysis of 269 horses from four populations and based on 14 microsatellite (MS) markers. Each genotyped horse is represented by a single vertical line divided into K colors, where K is the number of clusters assumed in each structure analysis. Each vertical bar represents an individual horse. The colors on each vertical bar represent the probability of the individual belonging to each cluster.

A Bayesian clustering method and population structure were used for clustering algorithms of multif-locus genotypes to identify the population structure and pattern of admixture within the populations. Population structure was used to estimate the number of groups that were classified by the group surveyed. The Bayes analysis set K values from 2 to 4 and examined the formation of colonies by group (Figure 5). The bar plot in Figure 5 confirms that the KGP and GOP populations showed different clusters from the other two Mongolian horse populations in all cases from the set K values. The remaining two populations showed similar patterns. The KGP and GOP populations were separated into different clusters when K3 and the KTP and USP populations were represented as a mixture with among. When the K value reached 4, the KGP and GOP populations were still separated into different clusters, but the KTP and USP populations primarily clustered with each other. Burn-in and MCMC repetitions (200,000 times and 1,000,000 times each, respectively) were used to estimate the optimal number of groups (ΔK values) when classifying groups between 2 and 4, which were repeated 10 times by setting the K values from 2 to 4, and the ΔK value was estimated using a structure harvester. The highest ΔK value (1.375%) was obtained from K value 3, which uses allele frequency data calculated with the 14 MS markers and distinguishes the three groups when the group is divided (Supplementary Table S2). This indicates that it is the most appropriate method.

## DISCUSSION

The purpose of this study was to verify the genetic diversity and correlation of Mongolian horses raised in five provinces using 14 MS markers. MS markers have been used to study the genetic diversity and population structure of horse breeds [23] and are commonly used in many countries for individual identification and parentage testing of horses.

The results of genetic variability, Nei’s DA genetic dis tance, PCoA, FCA, and population structure provided genetic evidence for the differentiation of the population, and polymorphism was observed in all provinces. A total of 160 alleles were detected in the 14 MS markers, the allele numbers ranged from 6 (HTG7) to 22 (ASB2), and the H_Obs_ values ranged from 0.561 (HTG4) to 0.868 (ASB17). ASB17 showed the highest H_Exp_ and PIC values of 0.885 and 0.866, respectively, whereas HTG4 showed the lowest H_Exp_ and PIC values of 0.572 and 0.535, respectively.

Our estimate of genetic diversity (H _Exp_ = 0.772, H_Obs_ = 0.757, and PIC = 0.737) in the Mongolian horse populations was found to be similar to that reported by Cho [24] (H_Exp_ = 0.773, H_Obs_ = 0.696, and PIC = 0.742). Tozaki et al [25] found that the average heterozygosity within each population of Mongolian horses was between 0.75 and 0.77. A higher value than our study was observed by Cho [26] (H_Exp_ = 0.809, H_Obs_ = 0.833, and PIC = 0.761) and Dorji et al [27] (H_Exp_ = 0.78, H_Obs_ = 0.79, and PIC = 0.77). Lower values were reported by Giacomoni et al [23] (H_Exp_ = 0.74, H_Obs_ = 0.628, and PIC = 0.706). The average number of alleles in our study was 11.43 in the 14 MS loci. This is higher than that reported by Cho [26], in which the MNA of the Mongolian horse populations using 11 MS loci was 8.18 alleles per locus. These MNA values were higher than the data published for other horse breeds and are similar to the study of Zuccaro et al [28], who observed an MNA of 10.09 alleles using 11 MS loci.

Nei’s DA genetic distance analysis highlighted the presence of a clear genetic separation between each province. The KTP and USP populations were the closest (DA = 0.0535), and the largest difference was calculated for the GOP and KGP populations (DA = 0.1797). The same results were obtained for PCoA, FCA, and the population structure. The KTP and USP populations had the closest genetic distances. This is possibly because they were each intercrossed with each other. In contrast, KGP and GOP populations were genetically differentiated from other provinces and suggest that both populations do not lose specific alleles unique. The population structure results also confirmed that the KGP and GOP populations were separated into different clusters in all cases.

According to Seo et al [29] and Dierks et al [30], MS markers are highly polymorphic when the sum of H_Exp_≥0.6 and PIC≥0.5. Therefore, the results of this study suggest that MS markers, except the HTG4 marker, can be used to aid the conservation, traceability, and improved abilities of the horse populations in Mongolia. Consequently, the Mongolian horses in this study showed a high level of genetic diversity. It is essential to secure genetic resources to analyze the genetic diversity of livestock and to identify their genetic relationships. While horses are economically important animals in Mongolia, only a few molecular genetic studies have been conducted on the genetic resources of Mongolian horses. Therefore, individual management of Mongolian horses based on genetic differences between them seems to be necessary. Finally, it is expected that this study can be used as basic data for the preservation and improvement of Mongolian horses and the establishment of breeds in the future.

## Figures and Tables

**Table 1:** Sample information in this study

Province types	Sample size	Collection location
Khentii province (KTP)	53	East Mongolia
Uvs province (USP)	43	West Mongolia
Omnogovi, Dundgovi province (GOP)	133	Southern Mongolia
Khovsgol province (KGP)	40	Northern Mongolia
Total	269	-

**Table 2:** Characterization of the 14 MS marker loci analyzed in 269 Mongolian horses

Marker	No of allele	H_Exp_	H_Obs_	PIC
AHT4	11	0.807	0.754	0.774
ASB17	20	0.885	0.868	0.866
ASB2	22	0.859	0.816	0.834
ASB23	15	0.822	0.838	0.794
CA425	12	0.798	0.786	0.764
HMS1	7	0.688	0.664	0.632
HMS2	12	0.796	0.825	0.758
HMS3	10	0.805	0.723	0.772
HMS6	8	0.799	0.810	0.762
HMS7	8	0.721	0.718	0.681
HTG4	7	0.572	0.561	0.535
HTG6	11	0.712	0.642	0.676
HTG7	6	0.716	0.748	0.660
VHL20	11	0.835	0.839	0.804
Mean	11.43	0.772	0.757	0.737

MS, microsatellite; H_Exp_, expected heterozygosity frequency; H_Obs_, observed heterozygosity frequency; PIC, polymorphism information content.

**Table 3:** The statistical analysis of MNA, heterozygosity, and PIC using the 14 MS markers in each province

Province types	MNA	H_Exp_	H_Obs_	PIC
Khentii province (KTP)	8.57	0.787	0.789	0.751
Uvs province (USP)	9.07	0.779	0.756	0.744
Omnogovi, Dundgovi province (GOP)	10.21	0.774	0.744	0.746
Khovsgol province (KGP)	7.07	0.751	0.738	0.706
Mean	8.73	0.773	0.757	0.737

MNA, mean number of alleles; PIC, polymorphism information content; MS, microsatellite; H_Exp_, expected heterozygosity frequency; H_Obs_, observed heterozygosity frequency.

## SUPPLEMENTARY MATERIAL

**Supplementary Table S1**. Nei’s genetic distance (DA) values among the four Mongolian horse populations

**Supplementary Table S2**. The mean likelihoods of models and standard deviation, and Delta K value using the Evanno method
