# Supplementary material for: Genetic diversity and population structure of Mongolian regional horses with 14 microsatellite markers
Source: Anim Biosci. 2022 Mar 2;35(8):1121–8. doi: 10.5713/ab.21.0497 (PMC9262727; doi:10.5713/ab.21.0497)
Supplement: Supplementary file 1 [file ab-21-0497-suppl.pdf]

Supplementary Table S1. Nei's genetic distance (DA) values among the four Mongolian horse populations

	Province types			
	KTP	USP	GOP	KGP
KTP	-			
USP	0.0535	-		
GOP	0.1022	0.1188	-	
KGP	0.1258	0.1154	**0.1797**	

KTP, Khentii province; USP, Uvs province; GOP, Omnogovi and Dundgovi province; KGP, Khovsgol province.

Supplementary Table S2. The mean likelihoods of models and standard deviation, and Delta K value using the Evanno method

K	Reps	Mean LnP(K)	StdevLnP(K)	Ln' (K)	|Ln''(K)|	Delta K
2	10	-13462.080000	0.198886	-	-	-
3	10	-13311.740000	24.583382	150.340000	33.790000	1.374506
4	10	-13195.190000	1.234189	116.550000	-	
